# Supplementary material for: Dynamic transcriptomic profiles of zebrafish gills in response to zinc supplementation
Source: BMC Genomics. 2010 Oct 11;11:553. doi: 10.1186/1471-2164-11-553 (PMC3091702; doi:10.1186/1471-2164-11-553)
Supplement: Additional file 2 — Interactive Direct Interaction Network representing the molecular interactions between zinc, copper, iron, calcium and proteins encoded by transcripts changed by zinc supplementation. Mini web-site containing index.html and hyperlinked pages in subdirectory describing a Direct Interaction Network automatically generated based on curated interactions contained within the proprietary PathwayArchitect database. Ovals represent proteins and the circles symbolize metal ions. Objects are coloured by their abundance in zebrafish at the time-point they were significantly different from the control is a scale from -4 fold (dark green) to +4 fold (dark red). Where significant differences were found at more than one time-point, the colour overlay shows expression at the first instance. Dark blue squares denote 'binding', and light blue squares 'expression'; green squares stand for 'regulation', green diamonds for 'metabolism', and green circles for 'promoter binding'. Arrow heads indicate directionality of the interaction where annotated. All nodes and edges can be further interrogated by selecting the relative area of the image. [file 1471-2164-11-553-S2.zip › PathwayArchitect Zn xs DIN/493475.html]

# BINDING:

|  |  |
| --- | --- |
| Type | BINDING |
| Effect | None |


---

|  |  |
| --- | --- |
| Score | 0 |


---

|  |  |
| --- | --- |
| Reference Count | 9 |


---

|  |  |
| --- | --- |
| Mechanism | Unknown |


---

|  |  |
| --- | --- |
| Reference:0 || Sentence | "Zn2+ enhanced the binding of Ca2+/S100A1 to twitchin kinase fragments (Kd < 50 nM) in assays using a BIAcore biosensor by reducing the S100A1 off rate." |
| PMID | 9363763 |
| Year | 1997 |
| Species | Rat |
| Journal | Eur J Biochem |
| RefScore | 1 |
| Source | PArchNLP |
  |
|  |  |


---

|  |  |
| --- | --- |
 Reference:1 || Sentence | "The Ca(2+)-binding S100A1 protein displays a specific and high expression level in the human myocardium and is considered to be an important regulator of heart contractility." |
| PMID | 12804600 |
| Year | 2003 |
| Species | Mouse |
|  | Human |
| Journal | Biochem Biophys Res Commun |
| RefScore | 0 |
| Source | PArchNLP |
  ||  |  |


---

|  |  |
| --- | --- |
 Reference:2 || Sentence | "S100 beta, a Ca2+ binding astrocytic brain protein implicated in brain development and neurophysiology, has elevated levels in progressive neurodegenerative diseases, Down's Syndrome, and Alzheimer Disease." |
| PMID | 8946407 |
| Year | 1996 |
| Species | Mouse |
| Journal | Neurobiol Learn Mem |
| RefScore | 1 |
| Source | PArchNLP |
  ||  |  |


---

|  |  |
| --- | --- |
 Reference:3 || Sentence | "The purpose of this study was to evaluate the expression of the Ca(2+)-binding S100 proteins S100A1, S100A2, S100A3, S100A4, S100A6 and S100B in normal skin." |
| PMID | 9274623 |
| Year | 1997 |
| Species | Human |
| Journal | Br J Dermatol |
| RefScore | 0 |
| Source | PArchNLP |
  ||  |  |


---

|  |  |
| --- | --- |
 Reference:4 || Sentence | "The Ca(2+)-binding S100A1 protein displays a specific and differential expression in the human myocardium and is considered to be an important regulator of heart function." |
| PMID | 10924368 |
| Year | 2000 |
| Species | Human |
| Journal | Biochem Biophys Res Commun |
| RefScore | 0 |
| Source | PArchNLP |
  ||  |  |


---

|  |  |
| --- | --- |
 Reference:5 || Sentence | "The purpose of this study was to examine the expression of 4 newly available polyclonal antibodies against the human recombinant Ca(2+)-binding S100 proteins, S100A1, S100A2, S100A4 and S100A6, in cutaneous melanoma and to correlate these findings with the standard S100 staining as well as with the metastatic potential of the primary." |
| PMID | 9162739 |
| Year | 1997 |
| Species | Human |
| Journal | J Cutan Pathol |
| RefScore | 1 |
| Source | PArchNLP |
  ||  |  |


---

|  |  |
| --- | --- |
 Reference:6 || Sentence | The Ca2+-sensor protein S100A1 was recently shown to bind in vitro to synapsins, a family of synaptic vesicle phosphoproteins involved in the regulation of neurotransmitter release. |
| Year | 2004 |
| PMID | 15147519 |
| Species | Mouse |
|  | Human |
|  | Rat |
| Journal | J Neurochem |
| RefScore | 1 |
| Source | PArchNLP |
  ||  |  |


---

|  |  |
| --- | --- |
 Reference:7 || Sentence | Expression of S100C/A11, a member of the Ca(2+)-binding S100 protein family, also markedly increased after addition of TGF-beta 1. |
| Year | 2004 |
| PMID | 15205326 |
| Species | Human |
| Journal | Cancer Res |
| RefScore | 1 |
| Source | PArchNLP |
  ||  |  |


---

|  |  |
| --- | --- |
 Reference:8 || Sentence | The three-dimensional solution structure of Ca(2+)-bound S100A1 as determined by NMR spectroscopy. |
| PMID | 16169012 |
| Year | 2005 |
| Species | Human |
| Journal | J Mol Biol |
| RefScore | 0 |
| Source | PArchNLP |
  |


---

|  |  |
| --- | --- |
